# Accumulation of colicin M protein and its biological activity in transgenic lettuce and mizuna plants

**DOI:** 10.3389/fpls.2023.1271757

**Published:** 2023-10-23

**Authors:** Nataliia Shcherbak, Heike Prochaska, Kateryna Lystvan, Yelizaveta Prokhorova, Anatoli Giritch, Mykola Kuchuk

**Affiliations:** ^1^Department of Genetic Engineering, Institute of Cell Biology and Genetic Engineering of National Academy of Sciences (NAS) of Ukraine, Kyiv, Ukraine; ^2^Nomad Bioscience GmbH, Halle (Saale), Germany

**Keywords:** bacteriocins, colicin M, transgenic lettuce, transgenic mizuna, pathogenic *Escherichia coli*, STEC, EHEC, multi-drug resistance

## Abstract

Food-borne illnesses caused by pathogenic *Escherichia coli* strains, especially enterohaemorrhagic *E. coli* (EHEC), are a serious public health problem, as debilitating disease and even death from such food poisonings have been repeatedly reported. Colicin M (ColM), a non-antibiotic antimicrobial protein produced by some strains of *E. coli*, has shown promising activity in controlling multiple enteropathogenic strains of *E. coli* and related pathogens. As contaminated green leafy vegetables are a frequent source of pathogenic *E. coli* infections, we genetically modified (GM) two edible crops, lettuce (*Lactuca sativa* L.) and mizuna (*Brassica rapa* subsp. nipposinica var. laciniata), to stably express the ColM gene and assessed the antibacterial activity of tissue extracts from these plants against selected *E. coli* strains *in vitro*. Transgenic plants of these species were developed using *Agrobacterium*-mediated transformation with a vector containing the ColM-coding gene (*cma*) under the control of the 35S promoter. Western blot analysis of recombinant ColM protein was performed in selected transgenic plants to confirm *cma* gene expression and quantify ColM accumulation. Extracts of transgenic plants expressing ColM showed significant activity against two major strains of EHEC (O157:H7 and O104:H4) as well as *E. coli* strains resistant to beta-lactam- and carbapenem-class antibiotics. Importantly, the antibacterial activity persisted in several subsequent generations of transgenic lettuce and mizuna plants that stably expressed the ColM gene. In addition, our results also show that the antibacterial activity of dried (up to 40°C) biomass of transgenic plants remained stable without a decrease for at least three months.

## 1 Introduction

Food contamination by Shiga-toxin producing *Escherichia coli* (STEC) strains, especially their subset which can cause hemorrhagic colitis (enterohemorrhagic *E. coli*, EHEC), has repeatedly resulted in outbreaks of debilitating intestinal infections affecting the health of millions of people worldwide. *E. coli* of serotype O104:H4, which combines enteroaggregative and Shiga-toxin producing properties (EAEC/EHEC), was responsible for a large outbreak of gastrointestinal disease in multiple countries in Europe in 2011. The high virulence and multidrug resistance (MDR) of this uncommon *E. coli* strain led to the infection of almost 4,000 people and resulted in 54 deaths (Navarro-Garcia, 2014; Tietze et al., 2015). *E. coli* O157:H7 is currently the predominant STEC serotype causing severe illnesses with a low infectious dose (50–100 colony-forming units (CFU/g or mL) (Puligundla and Lim, 2022). Frequently the spread of these pathogenic bacteria is associated with the consumption of animal products because they are commonly found in the intestinal tract of cattle (Low et al., 2005; Munns et al., 2015). However, green leafy vegetables, which are usually eaten raw without heat treatment from cooking, can be contaminated in the field through improperly composted manure, or contaminated irrigation water or soil (Alegbeleye et al., 2018; Waltenburg et al., 2021; Xu et al., 2021). A multistate outbreak of STEC O157:H7 associated with the consumption of Romaine lettuce was reported in the USA in 2011 (Slayton et al., 2013). Subsequently, the U.S. Centers for Disease Control and Prevention (USCDC) reported that in 2018 and 2019, several large outbreaks of *E. coli* O157:H7 linked to Romaine lettuce resulted in 439 illnesses and 5 deaths (USCDC, 2020). In spite of these experiences and the implementation of mitigation measures that followed, contaminated fresh leafy vegetables have continued to cause foodborne disease outbreaks (Irvin et al., 2021; Waltenburg et al., 2021).

The food industry employs various sanitizing methods both for disinfection of food processing equipment and for reduction of common foodborne pathogens associated with fresh-cut vegetables and fruit products. Currently, thermal inactivation is one of the most effective methods for the destruction of pathogenic bacteria in food, but the method is not generally amenable to decontaminating green leafy vegetables or some types of animal feed. For such products, sodium hypochlorite (NaClO) solutions can be used, but their residues are potentially harmful, can impart an undesirable taste, and the process is not environmentally benign (Tomás-Callejas et al., 2011). Antibiotics are often used prophylactically to prevent the spread of pathogenic bacteria on farms and in livestock. In response to the significant increase of MDR bacteria, legislative measures have widely been taken to limit or eliminate the use of antibiotics, including in the form of feed additives for livestock (Urban-Chmiel et al., 2022). According to the U.S. Food and Drug Administration (FDA), medically important antimicrobial drugs approved for use in food-producing animals decreased by 38% since 2015, which was the peak year of sales (FDA, 2021). However, this is only the beginning of the implementation of measures to prevent the increase of MDR bacteria and the problem of the constantly growing number of bacteria resistant to widely used antibiotics, including drugs of last resort, remains relevant. Antibiotics used in food-producing animals are closely related to those used in human medicine and antibiotic-resistant pathogenic bacteria foreshadow a growing global health crisis. Numerous studies have confirmed that bacteria use both phenotypic and genetic strategies that ensure the emergence of antibiotic resistance (Urban-Chmiel et al., 2022). There is already evidence of bacterial resistance to highly effective antibiotics, including transmission among *E. coli* strains of the resistance gene for the antibiotic colistin, considered the antibacterial of last resort in the management of severe and life-threatening Gram-negative infections (Liu et al., 2016; Paveenkittiporn et al., 2017). Also of concern is the rapidly increasing prevalence of *Enterobacteriaceae* harbouring carbapenemases and becoming resistant to carbapenem-class antibiotics. The spread of carbapenem resistance has led the World Health Organization (WHO) to designate carbapenem-resistant enterobacteria (CRE) as «priority pathogens» that pose the greatest global threat to human health (Willyard, 2017; Sheu et al., 2019). Alternative management strategies are therefore urgently needed, especially to control Gram-negative bacterial pathogens.

The spread of bacterial infections caused by food contamination with pathogenic microorganisms could be effectively prevented by bacteriocins, which are non-antibiotic antimicrobial proteins produced by bacteria to kill closely related strains, presumably by affording producing strains a competitive advantage for resources in their ecological niche (Gabrielsen et al., 2014; Kumariya et al., 2019; Benitez-Chao et al., 2021). Due to their high species and strain specificity (i.e., narrow spectrum of activity), the use of bacteriocins as antimicrobial agents offers important advantages over current chemistries, including sparing beneficial bacteria and thus avoiding microbial imbalances in the gastrointestinal (GI) tract (Cleveland et al., 2001; Cavera et al., 2015). Further, bacteriocins are natively produced by bacteria resident in the GI tract of humans and other animals, underscoring their evolutionary importance as well as their safety.

Therefore, bacteriocins offer unique advantages as potential alternatives to antibiotics, especially for prophylactic use in both food and feed. Within the bacteriocin family, colicins are antibacterial proteins produced by certain strains of *E. coli* that are effective against pathogenic strains of the same or closely related species (Callaway et al., 2004; Chérier et al., 2021). Colicins can be recombinantly produced using several platforms, including various plant hosts. Several plant-produced colicins have been approved as GRAS (Generally Recognized as Safe) antimicrobial food processing aids by FDA (GRAS Notice (GRN) No. 593, 2015). Colicin M (ColM) is the only one colicin interfering with peptidoglycan biosynthesis. Targeting such an essential bacterial component potentially makes the ColM-like proteins “universal” antibacterial agents (Chérier et al., 2021). ColM was first produced using transient gene expression methods in *Nicotiana benthamiana* plants, a well-accepted standard species for recombinant protein manufacturing, as well as in edible species of plants such as spinach and leafy beet (Schulz et al., 2015; Stephan et al., 2017; Hahn-Löbmann et al., 2019). Among the various colicins evaluated, ColM had the broadest antimicrobial activity against the seven major foodborne enterohemorrhagic *E. coli* (EHEC) strains, including the O157:H7 serotype (Schulz et al., 2015; Hahn-Löbmann et al., 2019) and *Klebsiella pneumoniae* (Łojewska et al., 2020).

The goal of our work was to create genetically modified edible plants that could stably express recombinant ColM. Previously, ColM had been recombinantly expressed only in non-food species of plants, including transiently and transgenically in *N. benthamiana* (Hahn-Löbmann et al., 2019) and transgenically in *N. tabacum* (Łojewska et al., 2020). The ColM isolated from these plants retained its conformation and was fully active. In the present study, we report for the first time the successful development of stable transgenic green leafy vegetables, lettuce and mizuna, expressing ColM and confirm the *antibacterial activity of* plant extracts for several generations of transgenic plants. We also demonstrate that heat dried (up to 40°C) transgenic plant biomass retained antimicrobial activity without significant reduction for at least 3 months. Our results suggest that bacteriocin-expressing leafy vegetables that are often the vectors for environmentally acquired Gram-negative bacterial infections could be used instead as a functional food to help control foodborne illnesses.

## 2 Materials and methods

### 2.1 Vector construction and bacterial strains

The vector pNMD46772 containing the ColM-coding *cma* gene under the control of a 35S promoter and selective *bar* gene under the control of a nos promoter was transformed to *Agrobacterium tumefaciens* strain GV3101. The transformed bacteria were grown on selective LB (Bertani, 1951) agar medium containing rifampicin 50 mg/L, gentamicin 25 mg/L and kanamycin 100 mg/L. Colonies were confirmed to be transformed with vector pNMD46772 using PCR analysis with primers specific for the *cma* gene.

### 2.2 Plant transformation and regeneration

*Agrobacterium*-mediated transformation of lettuce ‘Odeskyj curling’; with the vector pNMD46772 containing the *cma* gene was carried out as described in Shcherbak et al. (2013). After 48 h of co-cultivation with diluted *Agrobacterium* suspension supplemented with 100 mM acetosyringone, explants were placed on modified B5 (Gamborg et al., 1968) regeneration medium (B5 medium salts with 2.5% sucrose, 3 mg/L kinetin, 0.5 mg/L NAA) supplemented with 5 mg/L phosfinothricin (PPT) for selection. As shoot appeared, each one was removed and placed on the MS (Murashige and Skoog, 1962) medium supplemented with 0.5 mg/L of indole-3-butyric acid for rooting. Well established plants were transferred into the soil and grown in a greenhouse.

For genetic transformation of mizuna, hypocotyls of 5-7-day-old seedlings were used as explants. After co-cultivation with *Agrobacterium*, the explants were cultivated on callus induction MS medium containing 2,4-D (2 mg/L) and BAP (0.5 mg/L) for one week. Further regeneration of shoots took place on a nutrient medium containing zeatin (2 mg/L), BAP (1 mg/L) and NAA (0.5 mg/L). PPT at concentrations of 5 mg/L was used for the first step of mizuna transformants selection and after two weeks of cultivation on the regeneration medium the PPT concentration was increased to 10 mg/L. Regenerated plants were also rooted, transferred to the soil and grown in a greenhouse.

### 2.3 PCR and RT-PCR

Genomic DNA was isolated from plant leaves using the CTAB protocol for isolating DNA from plant tissues. PCR was carried out in 20 μl reaction volume containing 50 ng DNA, 200 μM each of forward and reverse primers, 200 μM dNTPs and 1 U Taq DNA polymerase (Thermo Scientific, USA). Amplification was performed on a Mastercycler® personal (Eppendorf, USA). Sequences of the primers used were as follows:

for *bar* gene amplification.

bar_fwd 5’-ACATCGAGACAAGCACGGTC-3’.
bar_rev 5’-GCCAGAAACCCACGTCATGC-3’.

for *cma* gene amplification.

colM_fwd 5’-ATGGAAACCCTTACTGTGCACGCTC-3’.
colM_rev 5’-CCTCTTACCAGACTCTTTGATGTG-3’.

The products of the amplification (411 bp when using primers for *bar* gene and 1003 bp for *cma* gene) were separated on 1% (w/v) agarose gels.

RT-PCR was performed for previously selected transgenic plants to detect *cma* gene transcripts. Total RNA was prepared from 100 mg of transgenic plant leaf tissue using an GeneJET RNA Purification Kit (Thermo Fischer Scientific, Waltham, USA) and reverse-transcribed using a RevertAid First Strand cDNA Synthesis Kit (Thermo Scientific, USA) with oligo dT primers. Genomic DNA from RNA preparation was removed by DNase I (RNase-free) treatment for 30 min at 37°C. A negative control for each sample consisted of the same reaction mixture without reverse transcriptase. The obtained cDNA was used as a template for PCR with gene-specific primers.

### 2.4 Extraction of plant material for protein analysis

Leaf material of transgenic and non-transgenic plants was ground in liquid nitrogen and total soluble protein (TSP) extracts were prepared with 5 volumes of extraction buffer (50 mM HEPES pH7.0, 10 mM K acetate, 5 mM Mg acetate, 10% glycerol, 0.05% Tween-20, 300 mM NaCl). After incubation for 20 min on ice and centrifugation for 10 min at 13000 rpm at 4°C, supernatant was used for immunoblot analysis and determination of antimicrobial activity. Protein concentration in the extracts was determined by the Bradford method using Bio-Rad Protein Assay (Bio-Rad Laboratories, Hercules, USA) and bovine serum albumin (BSA) (Sigma-Aldrich, Saint Louis, USA) as a standard.

### 2.5 Drying of plant material and extraction of dried material

Plant leaf material was harvested, frozen in liquid nitrogen and stored at -80°C. After 262 days, part of the material was thawed and incubated for 1 day at 40°C in an oven for drying. For final drying, material was incubated at room temperature for an additional 10 days. Dried material was further stored at room temperature. Fresh and frozen plant material was extracted with 5 volumes of extraction buffer, while dried material was extracted with 20 volumes of extraction buffer.

To allow direct comparison of activities for fresh and dried plant material, the weight factor for normalization of different extraction volumes and the weight loss during drying process were calculated as follows: Weight factor = fresh weight/dry weight * 5 (vol fresh extraction)/20 (vol dry extraction).

### 2.6 Purification of colicin M

ColM was transiently expressed in *N. benthamiana* and purified according to Stephan et al. (2017) with minor modifications. For purification plant material was extracted with the buffer containing 20 mM citric acid (pH4.0), 20mM NaH_2_PO_4_ and 30 mM NaCl in a 5:1 (v/w) buffer:biomass ratio. Ground leaf material admixed with pre-chilled extraction buffer was incubated on ice for 10 min followed by centrifugation at 10,000 x g for 15 min at 22°C. The supernatant was filtered using Miracloth (Merck KGaA, Darmstadt, Germany) and mixed with 10 mg/ml diatomaceous earth (Sigma-Aldrich, Saint Louis, USA) followed by incubation of the filtrate for 20 min at room temperature and centrifugation for 15 min at 10,000 x g at 22°C. The resulting supernatant was filtrated using filter discs with a pore size of 8–12 µm and the final filtrate was used for further purification by column chromatography. The filtrate was loaded on a HiTrap™Capto™MMC (GE Healthcare, Munich, Germany) column equilibrated with extraction buffer. The column was washed in two steps. The first wash step was performed with extraction buffer. The second wash step was done using diluted (30%) elution buffer consisting of 50 mM Na_2_HPO_4_ (pH 7.84), 10 mM citric acid and 1 M NaCl. The elution of ColM was carried out in a linear gradient from 30 to 100% of elution buffer concentration. The eluted fractions were analyzed by SDS-PAGE and Coomassie staining using Instant Blue™ staining solution (Expedeon, San Diego, CA, USA). The colicin containing fractions were pooled and dialyzed overnight at 4°C against 20 mM Na_2_HPO_4_ (pH 7.5), 10 mM citric acid and 50 mM NaCl. After dialysis, purified protein was frozen in liquid nitrogen and finally freeze-dried by lyophilization using freeze dryer Alpha 1–2 LDplus (Martin Christ Gefriertrocknungsanlagen, Osterode am Harz, Germany). Purified ColM batch 12 was used as a reference standard for antimicrobial activity and in immunoblot analysis.

### 2.7 Immunoblot analysis

Protein-containing plant extracts were mixed 1:1 with 2xLaemmli buffer and denatured at 95°C for 10 min. The total proteins were separated by sodium dodecyl sulfate polyacrylamide gel electrophoresis (SDS-PAGE) using a 15% polyacrylamide gel under reducing conditions and then transferred to a PVDF membrane. The membrane was incubated with a rabbit polyclonal ColM antibody 30365 (diluted 1:5,000) or monoclonal ColM antibody 3F9 (diluted 1:2,000) overnight. Polyclonal ColM antibody was generated against purified full length ColM by BioGenes GmbH (Berlin, Germany). Monoclonal ColM antibody 3F9 was generated against purified full-length ColM by Fraunhofer Institute for Cell Therapy and Immunology (IZI, Halle (Saale), Germany). Secondary antibodies were IgG (whole molecule) peroxidase affinity isolated antibody (Sigma-Aldrich, Saint Louis, USA), anti-mouse (diluted 1:5,000) or anti-rabbit (diluted 1:10,000). Determination of ColM concentration in extracts was done semi-quantitatively by comparison of different amounts of extracts with known amounts of purified ColM batch 12 reference protein.

### 2.8 Antimicrobial activity determinations

Microbiological studies were carried out on two laboratory strains of *E. coli* (DH10B and XL1-Blue) and a set of reference strains (**Table 1**). The bacteria were incubated at 37°C in LB medium (Bertani, 1951).

**Table 1:** Reference *E. coli* strains used in activity determinations.

collection number/strain designation (origin)	serotype	origin
ATCC BAA-2326	O104:H4	Doenitz Prolab e. Kfr., Augsburg (distributed Microbiologics product; obtained from ATCC collection)
ATCC 35150	O157:H7	Doenitz Prolab e. Kfr., Augsburg (distributed Microbiologics product; obtained from ATCC collection)
NCTC13352		The National Collection of Type Cultures (NCTC), UK (Public Health England)
NCTC13919		The National Collection of Type Cultures (NCTC), UK (Public Health England)
ATCC BAA-2452		Microbiologics (#01242P)

Semi-quantitative determinations of antimicrobial bacteriocin activity by a spot-on-lawn soft-agar overlay assay were performed as described in Schulz et al. (2015). Specific activity was calculated by normalization of arbitrary colicin activity units to TSP concentrations determined via Bradford assay to exclude effects of different extraction efficiencies.

### 2.9 Determination of minimal inhibitory concentration

Each respective bacterial test culture was grown in LB medium until OD600 of 0.2 and diluted to approximately 2x10^3^ CFU/µl. 5 µl of diluted culture were spotted on LB medium supplemented with 0.75% (w/v) agar, 0.1 mg/ml BSA (AppliChem, Darmstadt, Germany) and a dilution series of purified ColM (10 µg/ml to 1 ng/ml). Plates were incubated for 16-20 h at 37°C before MIC readout. The MIC was defined as the lowest concentration of ColM that inhibited visible growth of the test culture.

## 3 Results

### 3.1 Generation and analysis of transgenic plants expressing colicin M gene

**Figure 1:**
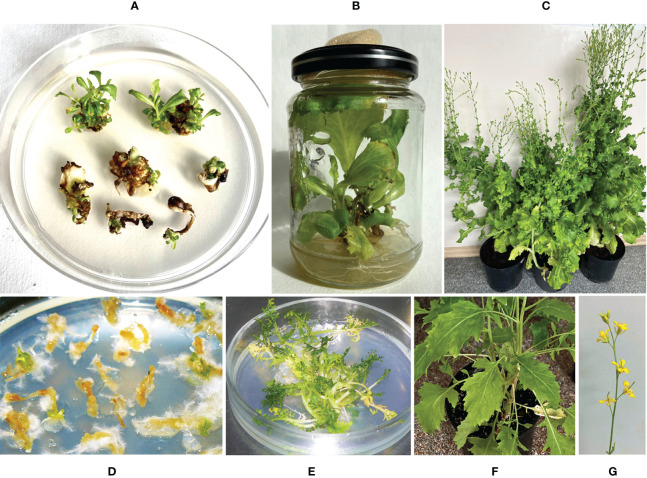
Generation of lettuce and mizuna transgenic plants expressing the gene of ColM. **(A)** *In vitro* lettuce plant regeneration from cotyledon explants after six weeks cultivation on selective medium; **(B)** rooting of regenerated lettuce shoots; **(C)** transgenic lettuce plants in a greenhouse; **(D, E)** *in vitro* regeneration of mizuna plants after four weeks **(D)** and 3 months **(E)** of cultivation on selective medium; **(F, G)** transgenic mizuna plants in a greenhouse.

We generated transgenic plants of lettuce (*Lactuca sativa* L.) ‘Odeskyj curling’ and mizuna (*Brassica rapa subsp. nipposinica* var. *laciniata*) using *Agrobacterium*-mediated transformation with a vector carrying the ColM-coding gene (*cma*). The vector pNMD46772 contains two expression cassettes: a *cma* gene under the control of a 35S promoter and the selective *bar* gene under the control of a nos promoter. Expression of the *bar* gene in transgenic plants provides for their resistance to the herbicide phosphinothricin (PPT). After five- or six-weeks incubation of lettuce cotyledons on the regeneration medium supplemented with the selective agent PPT (5 mg/L), green shoots were formed on the explants (**Figure 1A**). Regenerated plants developed roots after transfer to the medium supplemented with 0.5 mg/L of indole-3-butyric acid (**Figure 1B**). The resulting rooted lettuce plants were transferred to soil and grown in a greenhouse (**Figure 1C**). Mizuna plants were regenerated on the zeatin-containing media after 3-5 weeks of cultivation (**Figure 1D**). The selected plant lines resistant to 5 mg/L of PPT were cultivated under aseptic conditions (**Figure 1E**). Two lines were transferred to soil and grown in a greenhouse (**Figures 1F, G**). Greenhouse-maintained lettuce and mizuna plants bloomed and formed seeds in 3-4 months.

**Figure 2:**
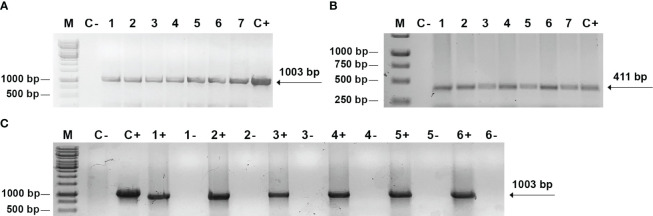
PCR and RT-PCR analysis of transgenic plants. **(A, B)** PCR analysis of genomic DNA. М *-* DNA mass ladder *O`GeneRuler™ 1 kb DNA Ladder (Thermo Fischer Scientific);* C- negative control with DNA from non-transformed plant; C+ - positive control, pNMD46772 plasmid DNA; 1-7 - independent transgenic lettuce (1-5) and mizuna (6-7) lines with primers to *cma* **(A)** and *bar* **(B)** genes; **(C)** RT-PCR analysis: C- negative control with RNA from non-transformed plant; C+ - positive control, pNMD46772 plasmid DNA; 1+ - transgenic mizuna RNA, 2+, 3+, 4+, 5+, 6+ - transgenic lettuce RNA; lanes 1-, 2-, 3-, 4-, 5-,6- - negative control for each corresponding sample, consisting of the same reaction mixture without reverse transcriptase.

All putative transgenic plants were initially screened for the integration of *bar* and *cma* genes using primers which should produce fragments of 411 bp and 1003 bp respectively. Amplification of the DNA of all plants revealed the expected fragments whose position is indicated by the arrows (**Figures 2A, B**). This result confirms the presence of *cma* and *bar* genes in the genome of the plants generated on the selective medium. A total of 5 independent transgenic lettuce plants and 2 mizuna plants were confirmed as PCR-positive for the transgenes integration. The purified RNA of transgenic plants along with wild type plants were used for cDNAs synthesis by reverse transcription. PCR product (1003 bp) obtained from the cDNAs by PCR with primers complementary to the *cma* gene confirmed ColM expression at the transcription level for transgenic plants of lettuce and mizuna (**Figure 2C**). A negative control for each sample consisted of the same reaction mixture without reverse transcriptase and no PCR products have been detected (lanes (1- to 6-), **Figure 2C**).

### 3.2 Transgenic lettuce and mizuna plants exhibit antibacterial activity against *E. coli*

**Figure 3:**
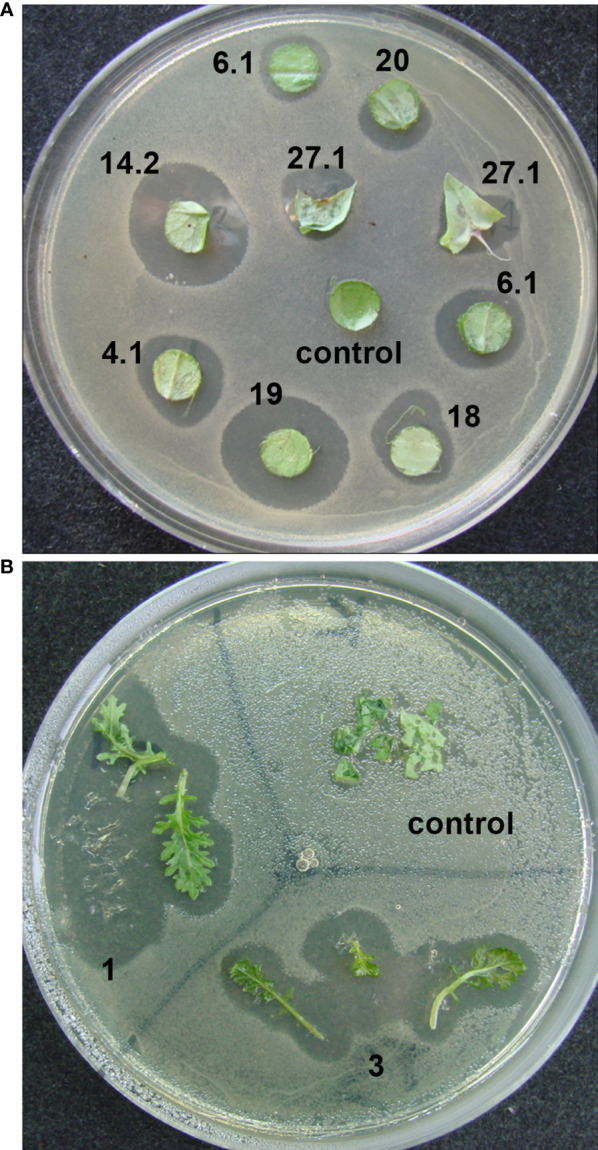
Determination of the antibacterial activity of ColM in transgenic lettuce and mizuna explants using a laboratory strain of *E. coli* XL1-Blue. **(A)** explants of different transgenic lines of lettuce; **(B)** leaves of two different transgenic lines of mizuna (BrT0#1, BrT0#3).

In an initial screen for antibacterial activity, the transgenic plants were tested by placing explants on agar medium covered with diluted bacterial suspension. Clear bacterial growth inhibition zones were observed around the explants of transgenic lettuce plants grown in a greenhouse (**Figure 3А**) and two transgenic mizuna plant lines maintained *in vitro* (**Figure 3B**).

**Figure 4:**
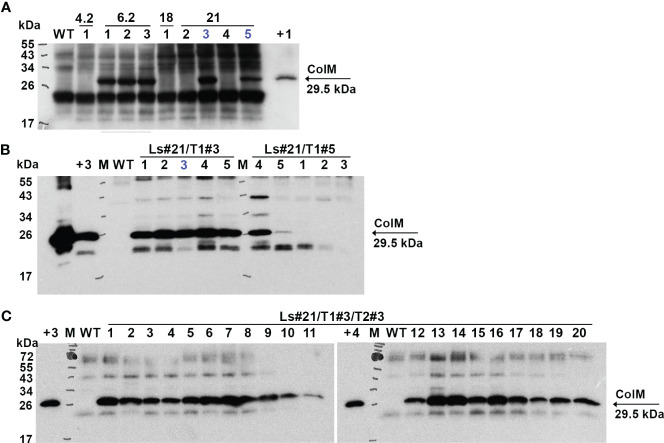
Expression of recombinant ColM detected by іmmunoblot in T1 **(A)**, T2 **(B)** and T3 **(C)** generations of transgenic lettuce (Ls46722). Transgenic or wild-type (WT) leaf material was extracted with 5 volumes of buffer. 7.5 µl of TSP were resolved using SDS-PAGE (15% gel) and expression was analyzed by immunoblot with polyclonal ColM antibody. **(А)** M - molecular weight marker; WT - wild-type non-transgenic lettuce plant; track numbers (1-5) correspond to different lettuce plants of the T1 generation of transgenic lines 4.2; 6.2; 18; 21 (indicated in the top row); +1 - positive control: 80 ng *N. benthamiana* TSP extract after *Agrobacterium*-mediated transient expression of ColM; blue color indicates plants which progeny analysis shown in **(B)**. **(B)** Track numbers correspond to different lettuce plants of the T2 generation of transgenic line 21; +2 - positive control: 1 µg purified ColM; +3 - positive control: TSP extract of Ls#6.2/2; blue color indicates plant which progeny analysis is shown in **(C)**. **(C)** Track numbers (1-20) correspond to different lettuce plants of the T3 generation of transgenic line 21 (Ls#21/3/3); +4 - positive control: 38 ng purified ColM.

Antimicrobial activity was also evaluated semi-quantitatively using a soft-agar overlay assay with plant extracts based on the highest dilution of each that caused a clearing effect. The results of these experiments are presented in **Table 2**. The data showed very comparable levels of antibacterial activity in different transgenic lines and even between the two analyzed species. However, a decrease or complete absence of antibacterial activity in some plants of T1 generation was also noted. The antibacterial activity of two T1 generation plants of the transgenic lettuce line Ls#21 (Ls#21/T1#3 and Ls#21/T1#5) differed by almost 10 times (**Table 2**). This result was also confirmed by analysis of recombinant ColM expression detected using immunoblot: the lower antibacterial activity values correlated with less pronounced immunoblot signals (**Figure 4A**, these lines are marked in blue).

**Table 2:** Antibacterial activity of transgenic lettuce and mizuna plant extracts against *E. coli* strain DH10B.

Transgenic line	Protein concentration, mg/ml	Antibacterial activity, AU	Specific activity, AU/mg/ml
Ls#16	2.42	5800	2396
Ls#19	1.77	2600	1465
Ls#6.2/T1#1	n.d.	2600	n.d.
Ls#6.2/T1#2	n.d.	3800	n.d.
Ls#6.2/T1#3	n.d.	3800	n.d.
Ls#6.2/T1#4	1.41	3800	2699
Ls#21/T1#3	n.d.	1100	n.d.
Ls#21/T1#5	n.d.	150	n.d.
Br#1	n.d.	2600	n.d.
Br#3	n.d.	3800	n.d.
Br#1/T1#1	1.64	1100	670
Br#1/T1#2	2.04	0	0
Br#1/T1#3	1.77	2600	1467
Br#1/T1#4	1.00	510	512
Br#1/T1#5	1.27	2600	2044
Br#3/T1#1	2.24	512	229
Br#3/T1#3	2.03	2048	1011

n.d. – no data/not determined.

### 3.3 Antibacterial activity was retained in T2 and T3 generations of transgenic plants

The same correlation between the antibacterial activity of the extracts, determined by soft-agar overlay assay, and the intensity of the immunoblot signals was observed when analyzing plants of the T2 generation of transgenic lettuce. **Table 3** summarizes results of analysis of the transgenic trait associated with ColM gene expression in the T2 generation of lettuce plants.

**Table 3:** The antibacterial activity and expression of the ColM detected by immunoblot in the T1 and T2 generations of transgenic lettuce.

Lettuce transgenic plants T1 generation	Antibacterial activity, AU	Lettuce transgenic plants T2 generation	Protein concentration, mg/ml	Antibacterial activity, AU	Specific activity, AU/mg/ml	WB signal
Ls#6.2/T1#1	2600	T2#1	1.50	3800	2541	+++
		T2#2	1.07	2600	2420	+++
		T2#3	1.28	2600	2034	++
		T2#4	1.33	5800	4346	+++
		T2#5	1.08	2600	2413	+++
Ls#6.2/T1#2	3800	T2#1	1.35	10	7	–
		T2#2	1.30	1700	1304	+++
		T2#3	1.23	225	183	+
		T2#4	0.89	2600	2909	+++
		T2#5	1.26	340	271	+
Ls#6.2/T1#3	3800	T2#1	1.35	3800	2818	+++
		T2#2	1.16	3800	3286	+++
		T2#3	1.23	3800	3097	+++
		T2#4	1.02	3800	3712	+++
		T2#5	1.07	3800	3555	+++
Ls#21/T1#3	1100	T2#1	1.30	2600	1999	+++
		T2#2	1.04	3800	3661	+++
		T2#3	0.87	2600	2994	+++
		T2#4	1.32	5800	4383	+++
		T2#5	1.04	2600	2512	+++
Ls#21/T1#5	150	T2#1	1.29	10	8	–
		T2#2	1.14	1	1	–
		T2#3	1.07	150	141	–
		T2#4	1.29	1100	855	+++
		T2#5	1.15	340	297	+

These results show that lettuce transgenic line Ls#6.2 retained significant antibacterial activity in almost all plants of the next generations T1 and T2. Only three plants of T2 generation of Ls#6.2/T1#2 line showed very low or no antibacterial activity (**Table 3**). One of the plants of T1 generation of lettuce line Ls#21 (namely Ls#21/T1#3) had high antibacterial activity as did all of its T2 descendants (**Table 3**; **Figure 4B**). Another T1 plant of line Ls#21 (Ls#21/T1#5) had low antibacterial activity and so did most of its offspring, except line Ls#21/T1#5/T2#4 (**Table 3**; **Figure 4B**). This line showed high antibacterial activity despite its parental line showing low activity. This observation could be the result of silencing of the ColM gene in transgenic line Ls#21/T1#5 (T1 generation) and the reversion of silencing in one of the descendants. In addition, the offspring of Ls#21/T1#3/T2#3 was analyzed for ColM expression by immunoblot. All 20 analyzed plants of the T3 generation, which were selected on PPT-containing medium, showed ColM expression (**Figure 4C**).

**Figure 5:**
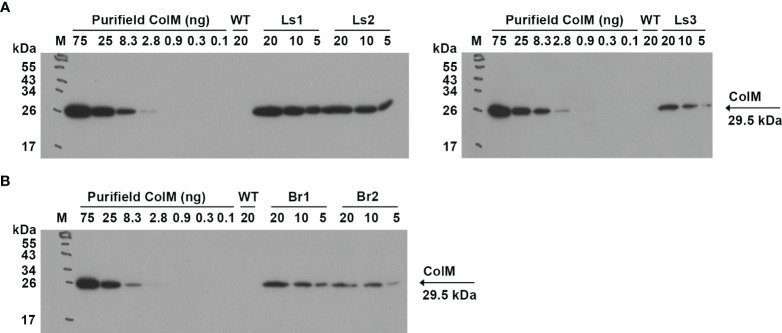
**(A)** Determination of ColM content in transgenic plant extracts by semiquantitative immunoblot analysis. Leaf material of ColM-expressing transgenic lettuce **(A)** and mizuna **(B)** plants was extracted with 5 volumes of buffer. The numbers above the tracks indicate the amount of purified ColM in ng or TSP extracts of transgenic plants in µl. Samples were resolved using SDS-PAGE (15% gel) and expression was analyzed by immunoblot with monoclonal ColM antibody. **(A)** M - molecular weight marker; WT - wild-type non-transgenic lettuce plant; Ls1 – transgenic lettuce plant of T2 generation Ls#6.2/T1#1/T2#12; Ls2 - transgenic lettuce plant of T2 generation Ls#21/T1#3/T2#16; Ls3 – transgenic lettuce plant of T1 generation Ls#16/T1#11. **(B)** M - molecular weight marker; WT - wild-type non-transgenic mizuna plant; Br1 – transgenic mizuna plant of T1 generation Br#1/T1#12; Br2 - transgenic mizuna plant of T1 generation Br#3/T1#13.

For lettuce and mizuna transgenic plants, we selected lines that had high antibacterial activity and calculated the concentration of recombinant ColM in total soluble protein (TSP) extracts generated by addition of 5 volumes of extraction buffer. The content of ColM in transgenic plant extracts (mg/ml) and fresh weight (FW) of plant biomass (mg/g) was calculated via antibacterial activity (**Table 4**) and by semiquantitative immunoblot analysis (**Figure 5**).

**Table 4:** Determination of ColM content in transgenic plant extracts by antibacterial activity and semiquantitative immunoblot analysis.

	ColM concentration, µg/ml extract		ColM concentration, µg/g FW	
	by activity	by WB	by activity	by WB
Ls#6.2/T1#1/T2#12	2.37 ± 1.75	1.06 ± 0.74	11.87 ± 8.76	5.28 ± 3.70
Ls#21/T1#3/T2#16	3.36 ± 2.50	2.08 ± 1.27	16.81 ± 12.50	10.40 ± 6.35
Ls#16/T1#11	2.72 ± 2.98	1.59 ± 0.87	13.61 ± 14.89	7.93 ± 4.33
Br#1/T1#12	2.46 ± 0.76	1.29 ± 0.80	12.28 ± 3.78	6.44 ± 3.99
Br#3/T1#13	0.54 ± 0.37	0.24 ± 0.26	2.70 ± 1.83	1.18 ± 1.28

The concentration was calculated in µg/ml extract and µg/g fresh weight (FW) of plant biomass and as a percentage of TSP and represented as an average value and standard deviation (AV ± SD) of three experiments.

### 3.4 Extracts of edible transgenic plants showed antimicrobial activity against pathogenic serotypes and multidrug-resistant strains

**Figure 6:**
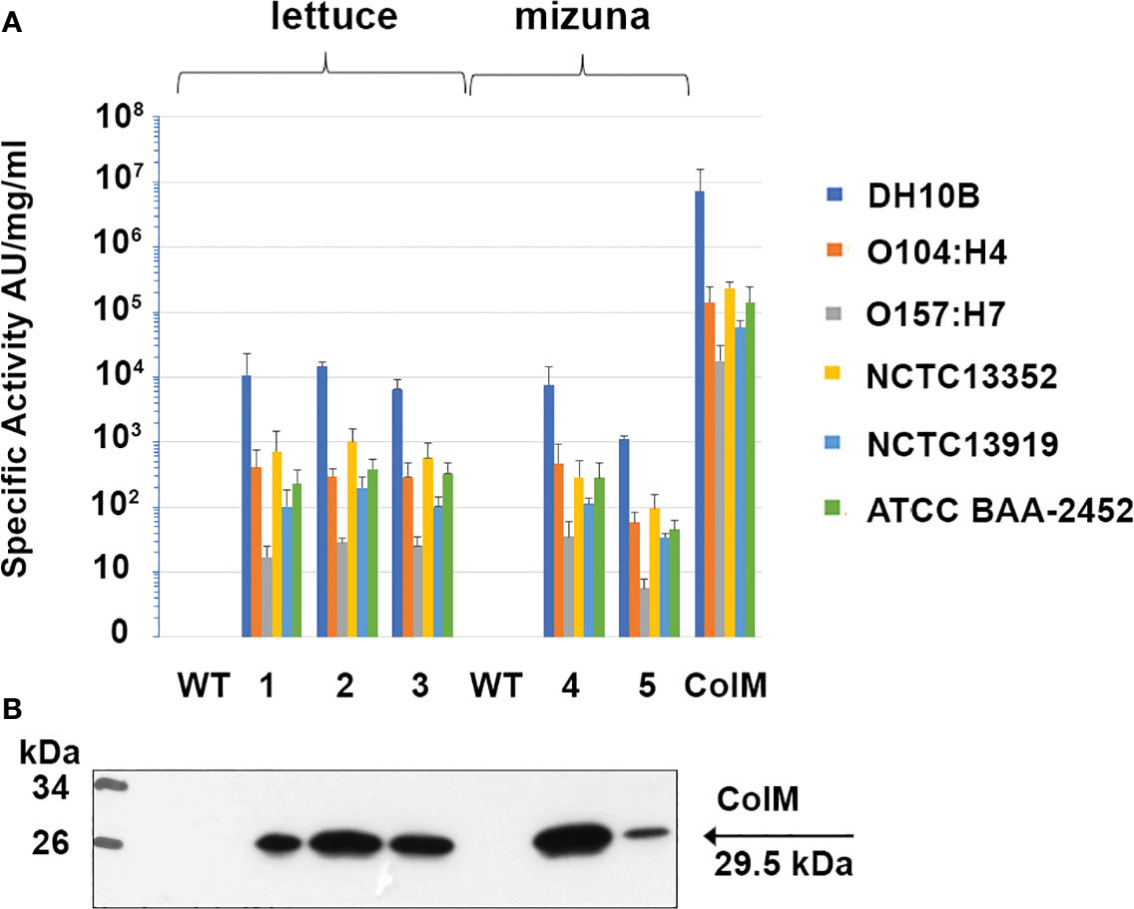
Antibacterial activity of ColM and ColM-containing transgenic plant extracts against *E. coli* strains O104:H4, O157:H7, O111:108, NCTC13352, NCTC13919 and ATCC BAA-2452. **(A)** Antimicrobial activity was determined semi-quantitatively on serial dilutions of TSP extracts by soft-agar overlay assay. Error bars indicate standard deviations of biological replicates (n = 3). AU- arbitrary colicin activity units. Transgenic lines: (1) Ls#6.2/T1#1/T2#12; (2) Ls#21/T1#3/T2#16; (3) Ls#16/T1#11; (4) Br#1/T1#12; (5) Br#3/T1#13). **(B)** Expression of recombinant ColM detected by іmmunoblot with monoclonal ColM antibody in the same transgenic lines. 7.5 µl of TSP extracts were resolved by SDS-PAGE (15% gel).

After finding high antibacterial activity in ColM-containing transgenic plant extracts against *E. coli* DH10B, we subsequently evaluated the activity of the extracts against a set of reference strains of *E. coli*, including two EHEC pathogenic serotypes (**Figure 6**). Аntibacterial activity against the emerging pathogenic serotype O104:H4 was higher than against the other EHEC serotype, O157:H7. In addition, ColM-containing extracts of edible plants, as well as purified ColM, also inhibited the growth of MDR *E. coli* strains. All three strains, including NCTC13352 that produces beta-lactamase and is therefore resistant to beta-lactam antibiotics, NCTC13919 that produces carbapenemases conferring resistance to carbapenems, and ATCC BAA-2452 that is resistant to both classes of these antibiotics, were sensitive to ColM-containing plant extracts.

Plant-derived purified recombinant ColM was used for determined Minimal Inhibitory Concentrations (MIC) for analyzed *E. coli* strains. The results showed that ColM was active against most of the *E. coli* strains used in the study with MIC values ranging from 4.0 to 78.0 ng/ml (**Table 5**). Only for *E. coli* strain ATCC 35150 O157:H7 the MIC of recombinant ColM was significantly higher (5 µg/ml). The MIC value serves as the basis for assessing the sensitivity or resistance of the pathogen to an antimicrobial agent. The data presented in **Table 4** show that the amount of ColM in transgenic lettuce plants extracts and mizuna line Br#1/T1#12 ranged from 1.06 to 3.36 µg/ml and these values are significantly higher than the MIC of purified recombinant ColM for the studied pathogenic bacterial strains (except for ATCC 35150 O157:H7). These results suggest the feasibility of using the obtained edible transgenic plants expressing ColM to help control foodborne disease.

**Table 5:** Antibacterial activity of colicin M against *E. coli* strains expressed as a minimal inhibitory concentration (MIC).

*E. coli* strain	MIC [ng/ml]
ATCC BAA-2326 O104:H4	78
ATCC 35150 O157:H7	5000
NCTC13352	19.5
NCTC13919	39
ATCC BAA-2452	78
DH10B	4

### 3.5 Antimicrobial activity in dried plant material does not decrease for at least 3 months

**Figure 7:**
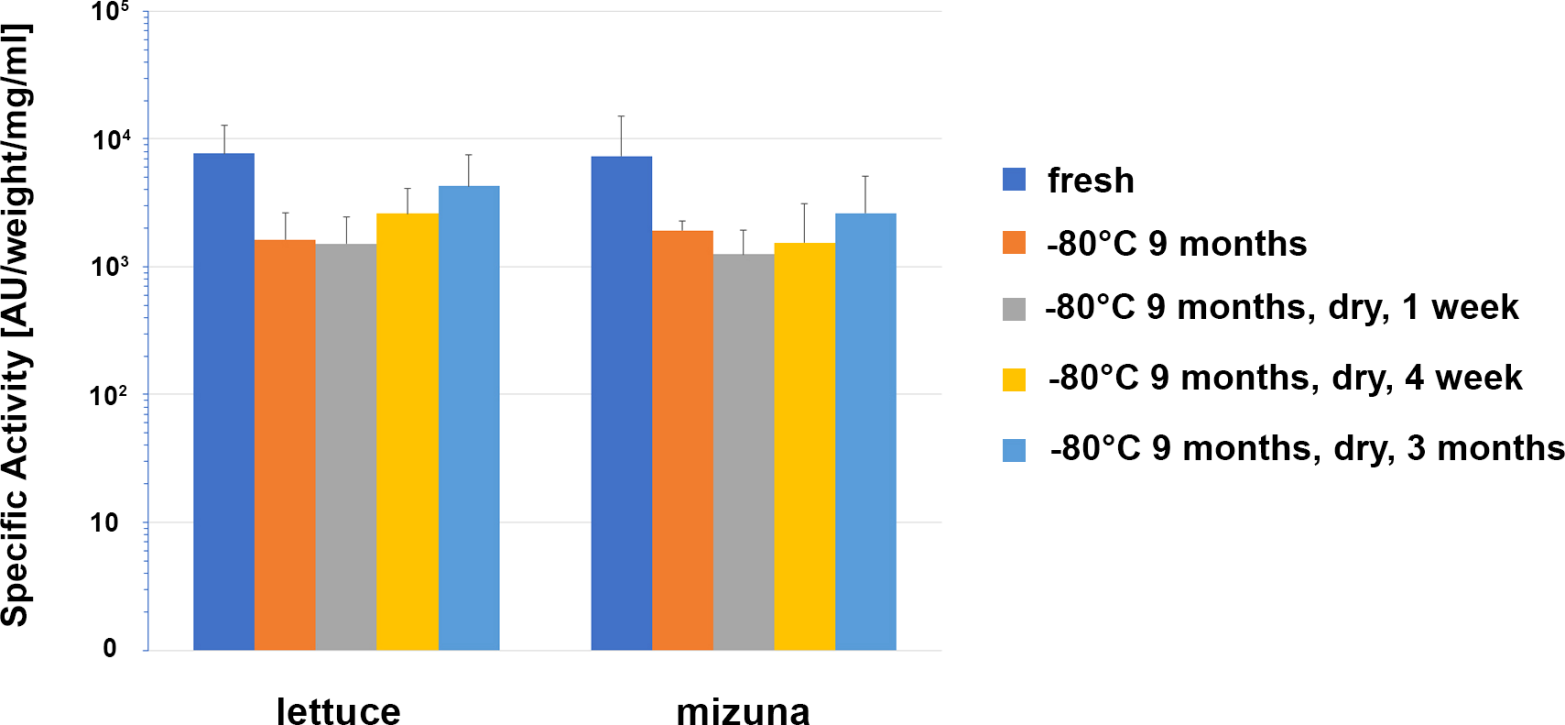
Comparison of the antibacterial activity of lettuce and mizuna samples under different storage conditions. Transgenic leaf material was extracted directly after harvest (fresh), after storage for 274 d at -80°C, and after storage for 262 d at -80°C followed by drying for 1 d at 40°C and subsequent storage for 1 week, 4 weeks or 3 months at room temperature. Activity of extracts was determined using soft-agar overlay assay against *E. coli* DH10B. In the graph, mean values with standard deviation of 6 different lettuce plants (Ls#6.2/T1#1/T2#11, Ls#6.2/T1#1/T2#12, Ls#21/T1#3/T2#15, Ls#21/T1#3/T2#16, Ls#16/T1#11, Ls#16/T1#12) or four different mizuna plants (Br#1/T1#11, Br#1/T1#12, Br#3/T1#11, Br#3T1#13) are shown. The concentration was calculated in μg/ml extract and μg/g fresh weight (FW) of plant biomass and as a percentage of TSP and represented as an average value and standard deviation (AV ± SD) of three experiments.

For edible plants producing pharmaceuticals, the issue of long-term storage conditions without loss of recombinant protein activity is highly important. Therefore, in our studies we also evaluated the antibacterial activity of dried samples of transgenic lettuce and mizuna plants expressing ColM. In these studies, leaf material stored at -80°C for 9 months was subsequently dried at 40° C and antibacterial activity was evaluated after 1 week, 4 weeks and 3 months of storage at room temperature. The results are shown in **Figure 7**.

Except for an initial drop in ColM antibacterial activity after 9 months of storage at -80°C, no additional activity decrease was observed that could be correlated to either the drying procedure or the length of time of additional storage. These results underscore the advantages of edible transgenic plants over other methods of recombinant ColM production. The levels of specific antimicrobial activity observed in plant extracts (**Figure 6**), and the stability of ColM under various storage regimes (**Figure 7**), suggest a new and inexpensive method for producing recombinant pharmaceutical products that does not require costly protein purification steps and is suitable for transportation and long-term storage without loss of activity.

## 4 Discussion

Plants have emerged as a reliable platform for production of recombinant proteins providing a safe and low-cost alternative to bacterial and mammalian cells systems. Transient expression in *Nicotiana benthamiana* has shown great potential for the manufacturing of recombinant biopharmaceuticals. Consequently, a wide range of medicinal proteins including recombinant vaccine antigens, monoclonal antibodies and other biotherapeutics have been synthesized in plants, some of which have advanced to clinical development (Daniell et al., 2009; Bendandi et al., 2010; Chichester et al., 2012; Gleba et al., 2014; Tusé et al., 2015; Ward et al., 2020; Leroux-Roels et al., 2022). The practice of using plants to produce valuable recombinant proteins has advanced significantly in recent decades (Ghequire and De Mot, 2017; Stander et al., 2022). With regard to antimicrobial proteins specifically, the cost of goods sold (COGS) for such proteins made by transient expression in plants at commercial scale was estimated to be comparable to those of products made by traditional food processing methods (McNulty et al., 2020). Despite the undoubted effectiveness of transient expression systems, the stable expression of antimicrobial proteins in transgenic edible plants makes it possible to reduce the costs of the most expensive part of production, namely, the extraction and purification of the recombinant protein (Łojewska et al., 2016). Such vegetables can be used as an antibacterial food or feed supplement without, or with minimal, downstream purification of the product.

The original idea of obtaining biopharmaceuticals in transgenic plants was implemented primarily for production of edible vaccines, and lettuce (*Lactuca sativa* L.) has often been used as a host plant for such studies (Matsui et al., 2009; Hamabata et al., 2019; Matsui et al., 2021). The main advantage of lettuce is that it is a fast-growing plant whose leaves are eaten raw and therefore can be used as an oral preparation. Lyophilized lettuce tissue has been used as a vehicle of oral vaccines against tuberculosis (Lakshmi et al., 2013), fasciolosis of cattle and sheep (Wesołowska et al., 2018) as well as to induce tolerance in hemophilia therapy (Su et al., 2015). The acceptance of lettuce plants for such studies is also explained by the fact that *in vitro* cultivation and genetic transformation techniques have been developed for lettuce, and it is also easy to grow indoors in the greenhouse, in hydroponic systems, and in the field. In addition, lettuce is a very popular crop, which is low in fat, calories and sodium, rich in fiber, iron, folate, and vitamin C and other health-beneficial bioactive compounds (Kim et al., 2016). Another plant in our study, mizuna, is also a fast-growing green leafy vegetable that is eaten raw, is high in phenolic compounds and flavonoids, especially hydroxycinnamic acids, and has high antioxidant activity (Khanam et al., 2012). Mizuna is a traditional and very popular crop in Asia and is increasingly attracting the attention of European and US consumers. Therefore, we developed mizuna plants expressing the ColM gene to impart antibacterial protective functions on a crop that is becoming well-accepted by consumers. In addition, and on a fundamental level, mizuna, in contrast to lettuce, has not been studied as a target for genetic transformation. To our knowledge, we are the first to report not only the successful genetic transformation of mizuna with the *cma* gene, but also the first genetic transformation of this plant species.

According to published data, the transient expression of ColM has been described in the standard host *Nicotiana benthamiana*, as well as in edible plant hosts such as spinach and leafy beets. Purified ColM obtained in plants by the transient expression method had the broadest antimicrobial activity against pathogenic strains of *E. coli* and, in mixtures, supplemented the effectiveness of other colicins (Schulz et al., 2015; Stephan et al., 2017). In addition, transgenic *N. benthamiana* (Hahn-Löbmann et al., 2019) and *N. tabacum* L. plants expressing ColM were developed (Łojewska et al., 2020) and the antibacterial activity of purified ColM obtained from these plants was confirmed against an array of pathogenic strains, including clinical isolates of *E. coli* and *Klebsiella pneumonia*. In the present study, we describe for the first time the antibacterial activity of edible transgenic plants stably expressing recombinant ColM. Our data demonstrate that ColM can be stably expressed in edible plants at a level that provides the desired antibacterial functionality. We also confirmed both the antibacterial activity of transgenic plant extracts and the inheritance of this trait in subsequent generations. Our studies once again confirm that ColM expressed in plants retains full functional activity against *E. coli* pathogenic serotype O104:H4 and O157:H7. Furthermore, extracts of edible plants containing ColM were found to inhibit the growth of multidrug-resistant strains of *E. coli* that produce beta-lactamases and carbapenemases. These results are of practical importance as carbapenems are considered to be antibiotics of last resort and antimicrobial options for the treatment of carbapenem-resistant *Enterobacteriaceae* (CRE) are limited (Sheu et al., 2019). Our results also show that ColM antimicrobial activity is retained for at least three months without loss of activity in heat-dried (up to 40°C) transgenic lettuce and mizuna biomass. According to published data, ColM is a fairly stable protein. It was demonstrated that pure bacteria-derived СolM remain stable for more than 1 year in solution at concentrations above 1 mg/ml (Schaller et al., 1981). Although the inactivation of ColM occurred when the solution was diluted, this process can be effectively slowed down or stopped by various additives to the medium. High stability was also demonstrated for plant-derived purified recombinant СolM. Recombinant plant-derived СolM remained stable in lyophilized form and in solution stored at 4°C. The solution stored at room temperature was less stable, but ColM was not inactivated for 8 weeks and showed the best stability of all 4 colicins tested (Hahn-Löbmann et al., 2019). However, proteolytic degradation of this bacteriocin is possible in the plant cell. We believe that dried plant biomass retained antimicrobial activity of ColM mostly because of drying procedure which has been applied. Slow drying of entire leaves prevented the decompartmentalization in plant cells thus avoiding massive attack of plant proteases against ColM which could happen, e.g., in case of grinding. In agriculture, dry plant biomass (hay) is known for a good stability of nutritional crude protein. Using a drying procedure for preservation and storage of ColM-containing leaf material was partially inspired by that knowledge. Indeed, this approach appeared to be productive and provided both stable and functional bacteriocine. To our knowledge, this is the first study that demonstrates the antibacterial activity of extracts of edible transgenic plants expressing ColM against various *E. coli* strains, including pathogenic and MDR pathotypes. The use of edible plants expressing ColM as a food or feed additive for preventive purposes is especially poignant due to the magnitude and recurrence of foodborne *E. coli* outbreaks worldwide, the role of leafy vegetables in their transmission, and the continued threat posed by MDR bacteria due to the overuse of antibiotics in agriculture.

## Data availability statement

The original contributions presented in the study are included in the article/supplementary material. Further inquiries can be directed to the corresponding author.

## Author contributions

NS: Investigation, Writing – original draft. HP: Data curation, Investigation, Writing – original draft. KL: Investigation, Writing – review & editing. YP: Investigation, Writing – review & editing. AG: Conceptualization, Writing – review & editing. MK: Conceptualization, Supervision, Writing – review & editing.
